# Delayed Improvement of Depression and Anxiety after Transcatheter Aortic Valve Implantation (TAVI) in Stages of Extended Extra-Valvular Cardiac Damage

**DOI:** 10.3390/jcm10081579

**Published:** 2021-04-08

**Authors:** Laura Bäz, Marisa Puscholt, Claudia Lasch, Mahmoud Diab, Sven Möbius-Winkler, P. Christian Schulze, Gudrun Dannberg, Marcus Franz

**Affiliations:** 1 Department of Internal Medicine I, Jena University Hospital, Friedrich Schiller University, 07747 Jena, Germany; Laura.Baez@med.uni-jena.de (L.B.); m.puscholt@web.de (M.P.); Claudia.Lasch@med.uni-jena.de (C.L.); Sven.Moebius-Winkler@med.uni-jena.de (S.M.-W.); Christian.Schulze@med.uni-jena.de (P.C.S.); Gudrun.Dannberg@med.uni-jena.de (G.D.); 2 Department of Cardiothoracic Surgery, Jena University Hospital, Friedrich Schiller University, 07747 Jena, Germany; Mahmoud.Diab@med.uni-jena.de

**Keywords:** aortic stenosis, staging of extra-valvular cardiac damage, depression, anxiety, transcatheter aortic valve implantation, follow-up

## Abstract

Background: Depression and anxiety are frequently occurring and likely to be linked to the severity of cardiac diseases like aortic stenosis (AS). This seems to be of interest since a staging classification of extra-valvular cardiac damage in AS has been introduced and shown to be of prognostic relevance. Objective: The current study aimed to investigate the frequency of depression and anxiety in association to staging and their dynamics after transcatheter aortic valve implantation (TAVI). Methods: A total number of 224 AS patients undergoing TAVI were classified according to the 2017 staging classification into stage 0 to 4 and further dichotomized into group A (stage 0 to 2) and B (stage 3 and 4). Using the Hospital Anxiety and Depression Scale (HADS-D), patients were assigned to depressive versus non-depressive or anxious versus non-anxious per staging group respectively, and analyzed at baseline, 6 weeks, 6 months and 12 months after TAVI. Results: After dichotomization, 158 patients (70.5%) were assigned to group A and 66 patients (29.5%) to group B. The part showing pathologic values for depression was 25.4% (57/224 patients) in the entire collective, 26.6% (42/158 patients) in group A and 22.7% (15/66 patients) in group B (*p* = n.s.). The proportion showing pathologic values for anxiety was 26.8% (60/224 patients) in the entire collective and did not differ between group A (24.7%, 39/158 patients) and B (31.8%, 21/66 patients) (*p* = n.s.). In patients revealing pathologic values for depression or anxiety prior to TAVI, there were significant and stable improvements over time observable already in short-term (6 weeks) follow-up in group A, and likewise, but later, in long-term (6/12 months) follow-up in group B. Conclusions: Although of proven prognostic relevance, higher stages of extra-valvular cardiac damage are not associated with higher rates of pre-existing depression or anxiety. The TAVI procedure resulted in a persisting reduction of depression and anxiety in patients showing pathologic values at baseline. Notably, these improvements are timely delayed in higher stages.

## 1. Introduction

Among the wide range of ageing-related cardiovascular diseases as the leading cause of death in older adults, acquired heart valve diseases, in particular degenerative aortic stenosis (AS) as the most frequently occurring variant, are of great clinical and health economic interest. The prevalence of AS is approximately 5% in patients of 75 years or older and in 50% of these cases, the disease presents in the symptomatic stage and therefore requires treatment [1]. Hitherto, besides addressing AS-related symptoms, there is no medical treatment of proven evidence with respect to slowing, stopping, or even reversing disease progression. Thus, the only causative therapy is aortic valve replacement, which can be realized surgically or by transcatheter approaches [2]. 

In the era before transcatheter aortic valve implantation (TAVI) was an established treatment strategy, up to one third of elderly patients were excluded from causative therapy due to comorbidities and a resulting high surgical risk [3]. In the last decade, the TAVI procedure underwent a rapid evolution from a treatment option for elderly high-risk patients to the therapy of choice in patients of 70 years or older irrespective of surgical risk [4,5,6,7,8,9,10,11,12]. Nevertheless, whenever treating AS patients by TAVI, one has to consider the specialty of this collective, in particular with respect to high subject ages and the multitude of comorbidities. Thus, besides functional outcomes or prognosis quo ad vitam, quality of life (QoL) and mental health aspects are of great importance when assessing the value of a therapy. In that context, Boureau and co-workers reported on significant improvements of physical activity but not mental well-being 6 months after TAVI in a typical patients’ collective. In contrast, when analyzing the subgroup of patients showing depressive symptoms prior to TAVI, a relevant improvement of mental health could be observed [13]. 

Studies explicitly focusing on depression and anxiety as mental co-morbidities of elderly patients suffering from cardiovascular diseases are rare until today, and there are nearly no reports on the potential impact of a defined interventional therapy treating the somatic disease on the severity of symptoms of the mental disorder. In a variety of studies dealing with heart failure, high rates of clinically significant depressive symptoms (up to 21.5%) and anxiety (up to 30%) were found, especially in elderly patients, and could be linked to unfavorable outcomes [14]. In patients suffering from coronary artery disease, the prevalence of depression has been reported to be up to 45%, with a severe impact on mental health and QoL. Moreover, depression in these patients could be identified as a risk factor for severe cardiovascular events [15,16,17,18]. Also, in patients undergoing cardiac surgery including conventional aortic valve replacement, depression and anxiety were proven to increase the risk of adverse events and complications in the postoperative course [19,20]. 

The precise question for the frequency and prognostic impact of anxiety and depression in a typical AS patients’ collective undergoing TAVI has not been answered concludingly until now. In a prospective analysis of the German Aortic Valve Registry (GARY) from 2016, QoL of TAVI patients was assessed using the EuroQol questionnaire EQ-5D-3L including the item “anxiety/depression”. Whereas the authors could outline relevant improvements of mobility and usual activities, for anxiety and depression, only minor effects were observable [21]. Another trial in that field focused on the association of depression with mortality in patients suffering from severe AS treated by either TAVI or surgical aortic valve replacement. Here, a very high frequency of depressive symptoms of 31.5% could be evidenced, whereas the rate of patients fulfilling diagnostic requirements for formal depressive disorder was not more than 8.6%. Presence of depression was shown to be associated with higher rates of short- and mid-term mortality and persistent depression 6 months after TAVI, with a 3-fold increase in 1-year mortality [22]. Thus, a high rate of unreported cases of depression in elderly AS patients has to be supposed. In a prospective observational study including 140 elderly real-world AS patients with moderate surgical risk that underwent TAVI, our group reported on high rates of anxiety (28.6%) and depression (23.6%) prior to TAVI using the German version of the Hospital Anxiety and Depression Scale (HADS-D). Interestingly, in the subgroup showing pathologic values for depression at baseline, there was a significant improvement of depression already 6 weeks after TAVI, remaining stable in long-term follow-up. Similar effects of TAVI in terms of anxiety reduction could be evidenced for patients showing pathologic values for anxiety at baseline. Possibly due to the small number of patients, we could not observe an association with 1-year mortality [23].

The majority of available studies on the prevalence and clinical impact of depression and anxiety in elderly cardiovascular disease patients describe heart failure cohorts including both heart failure with reduced and preserved ejection fraction [14]. Thus, one could hypothesize that the occurrence and severity of mental disorder symptoms might be linked to the extent of structural and functional alterations of the heart muscle and thereby heart failure severity. This seems to be of special interest since a novel staging classification of the extent of extra-valvular cardiac damage in AS, as a disease continuum beginning with left and resulting in right heart failure, has been introduced recently. In the PARTNER 2 trials collective including 2703 patients, higher stages showed a clear correlation to a worse prognosis [24]. 

Against that background and since high prevalence rates of both depression and anxiety have been reported not only in heart failure but also in elderly AS patients [21,22,23], the aim of the current study was to prospectively investigate a real-world collective of TAVI patients, which has been classified according to the staging classification of extra-valvular cardiac damage [24] at baseline, prior to TAVI. Thereby, we aimed to describe prevalence rates of depression and anxiety in association to 2 predefined staging groups (group A—left heart damage, group B—pulmonary hypertension/right heart damage) to test whether the extent of structural or functional alterations of the heart impacts the frequency of depression/anxiety occurrence, and to analyze longitudinally whether patients in the different staging groups show different dynamics in terms of depression/anxiety development after TAVI.

## 2. Material and Methods

### 2.1. Study Cohort

A total of 224 patients who underwent TAVI due to severe degenerative aortic valve stenosis (AS) in the symptomatic stage were included in this prospective observational study, which was approved by the local ethics committee of the University Hospital Jena (Jenaer Aortenklappenregister—JAKR; registration number: 4815-06/16). All patients gave written informed consent for participation in the study. In addition to routine clinical, laboratory, functional and imaging analyses, patients were screened with respect to the presence of anxiety and depression. Follow-up was carried out 6 weeks, 6 months and 12 months after TAVI in our outpatient department. For two subgroups of patients, namely the depressive and anxious subgroup of patients assigned to staging group B, as described below, a so-called ‘long-term follow-up’ was defined as a fusion of the timepoints ‘6 months’ and ‘12 months’ after TAVI due to relatively small patient numbers in these groups. In general, the longest available follow-up timepoint in each subgroup has been implemented in the analyses. Patients that experienced severe peri-procedural complications, which are very rare in our local registry, were excluded from the study. Baseline characteristics of the entire study collective as well as the different staging groups are summarized in Table 1.

### 2.2. Acquisition of Depression and Anxiety, Quality of Life and Health Status

For the assessment of depression and anxiety, the German version of the Hospital Anxiety and Depression Scale (HADS-D), enabling clear and reliable discrimination between anxiety and depression as well as somatic symptoms [25], was available. It implies 14 items assignable to two subclasses (anxiety and depression), with 7 items for each subclass, and is capable to record anxiety and depression states in a hospital setting [26]. As a detection threshold for depression and/or anxiety, a cut-off point ≥8 was defined according to the literature [27]. 

QoL measurements were performed using the current version of the EuroQol questionnaire (EQ-5D-5L), which is an accepted tool for the standardized simple generic QoL assessment already used in TAVI patients [28,29]. In this study, focusing on the presence of depression and anxiety in association with extra-valvular cardiac damage, we only consider the item EQ-5D-5L_anxiety/depression in our analyses. 

### 2.3. Assessment of the Level of Extra-Valvular Cardiac Damage According to the 2017 Staging Classification

All patients included in the current study were assigned to stages 0 to 4 at baseline in adherence to the criteria of the staging classification recommended by Généreux and co-workers in 2017 (stage 0: no cardiac damage, stage 1: left ventricular (LV) damage (increased LV mass index >115 g/m^2^ for males and >95 g/m^2^ for females, E/e’ > 14, LV ejection fraction 34 mL/m^2^), stage 2: moderate to severe mitral regurgitation, atrial fibrillation, stage 3: pulmonary vasculature or tricuspid damage (systolic pulmonary hypertension ≥ 60 mmHg, moderate to severe tricuspid regurgitation), stage 4: right ventricular damage (moderate to severe right ventricular dysfunction)) [24]. To allow reasonable analysis, in particular with respect to statistical limitations due to low patient numbers per group when operating with 5 groups, we further dichotomized patients into staging group A (stages 0 to 2) and staging group B (stages 3 to 4).

### 2.4. Statistics

Statistical analyses were performed using SPSS statistical software, version 25.0 (IBM SPSS Statistics for Windows. Armonk, NY, USA). For categorical variables, data are expressed as numbers or percentages, where appropriate. Continuous variables are given as mean ± standard deviation (SD). The Kruskal–Wallis or the Mann–Whitney U test were performed to test for statistically significant (*p*-value < 0.05) differences between the groups.

## 3. Results

### 3.1. Characterization of the Study Population and Assignment to the Staging Classification of Extra-Valvular Cardiac Damage

**Figure 1:**
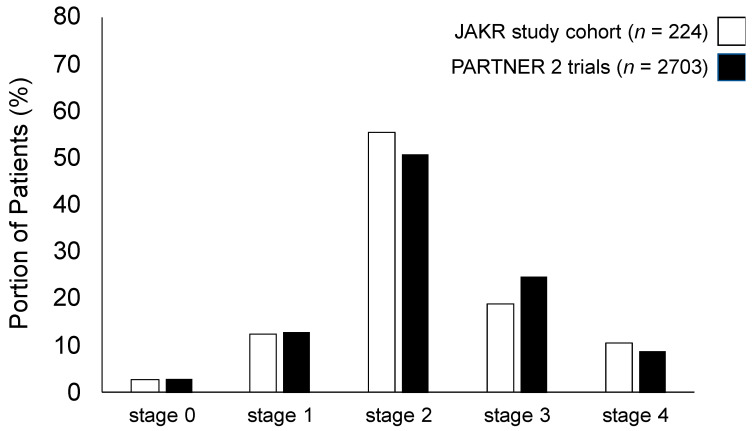
Comparison of extra-valvular cardiac damage staging according to the 2017 staging classification in the Jenaer Aortenklappenregister (JAKR) study cohort presented here (*n* = 224, light grey) compared to the PARTNER 2 trials cohort (*n* = 2703, dark grey) showing high levels of consistency.

The 224 patients included in our study represent a typical collective of elderly patients undergoing TAVI due to severe degenerative AS in the symptomatic stage exhibiting moderate surgical risk. The mean age was 77.9 ± 7.5 years, 45.1% were male and the mean Society of Thoracic Surgeons (STS)-Score was 4.4% ± 3.1%. The study collective was categorized as described in the material and methods section according to the staging classification of extra-valvular cardiac damage [24] into stages 0 to 4. The distribution of the different stages was as follows: stage 0: 2.7% (6/224 patients), stage 1: 12.5% (28/224 patients), stage 2: 55.4% (124/224 patients), stage 3: 18.8% (42/224 patients), stage 4: 10.7% (24/224 patients). Comparison of these percentages with the results of the staging group distribution in the PARTNER 2 trials, in which the classification was validated and shown to be of prognostic relevance, shows a high level of consistency (Figure 1). Referring to the relatively small number of subjects in some staging groups, especially in stages 0 and 4, we decided to further dichotomize patients into staging group A and B as described above, with group A summarizing AS patients with left heart damage (*n* = 158) and group B such with additional pulmonary hypertension/right heart damage (*n* = 66). The baseline characteristics of both the entire patient’ collective and these staging groups are given in Table 1.

### 3.2. Assessment of Depression and Anxiety Prior to TAVI 

In the entire patient cohort (*n* = 224), the mean values for depression and anxiety assessed by HADS-D were 5.4 ± 3.7 (HADS-D_depression) and 5.7 ± 3.5 (HADS-D_anxiety). Using a cut-off value of ≥8 points for depression and/or anxiety in the HADS-D, there were the following frequencies of patients showing pathologic values at baseline. Considering the entire patients’ collective (*n* = 224), 25.4% (*n* = 57/224) showed pathologic values for depression and 26.8% (*n* = 60/224) for anxiety. With respect to the predefined staging groups, in group A, 26.6% (*n* = 42/158) of patients showed pathologic values for depression (HADS-D_depression: 10.1 ± 2.4) and 24.7% (*n* = 39/158) for anxiety (HADS-D_anxiety: 10.3 ± 1.9). In group B, 22.7% (*n* = 15/66) of patients showed pathologic values for depression (HADS-D_depression: 10.9 ± 2.7) and 31.8% (*n* = 21/66) for anxiety (HADS-D_anxiety: 10.4 ± 2.3). The frequencies of depression and anxiety did not show significant differences between the staging groups (*p* = 0.547 for depression and *p* = 0.273 for anxiety). The results are given in Table 1. Moreover, for EQ-5D-5L_anxiety/depression (1.7 ± 0.9 in group A vs. 2.1 ± 1.1 in group B, *p* = 0.017) and regarding brain natriuretic peptide (BNP)-levels (483 ± 574 pg/mL in group A vs. 1480 ± 2444 pg/mL in group B, *p* < 0.001), there were significant differences between the staging groups.

### 3.3. Dynamics of Depression and Anxiety in Short- and Long-Term Follow-Up after TAVI 

When including the entire patients’ collective, there were no significant changes concerning both depression and anxiety measured by HADS-D, between the different follow-up time-points, each compared to baseline (*p* = n.s.). Nevertheless, a significant improvement at the 6-week follow-up compared to baseline could be observed for EQ-VAS (63.4 ± 17.2 vs. 57.6 ± 18.2, *p* = 0.001), BNP (370 ± 582 vs. 790 ± 1502, *p* < 0.001) and six minutes walk test (SMWT) (288 ± 129 vs. 202 ± 160, *p* < 0.001). These changes presented stable even at the 6-month as well as the 12-month follow-up (each *p* < 0.05 compared to baseline). For EQ-5D-5L_anxiety/depression, we could observe a significant decrease at the 6-month follow-up (1.5 ± 0.8, *p* = 0.006) compared to baseline (1.8 ± 1.0), which was not present anymore at the 12-month follow-up (1.6 ± 0.9, *p* = n.s. compared to baseline). 

### 3.4. Predefined Subgroups According to Depression and Anxiety (HADS-D) in the Entire Collective

To test whether pre-existing depression or anxiety is influenced by TAVI, we divided the study population (*n* = 224) into different subgroups according to HADS-D using a cut-off ≥8 points for depression (denominated ‘depressive subgroup’) or anxiety (denominated ‘anxious subgroup’) defining pathologic and non-pathologic values, as described above.

**Figure 2:**
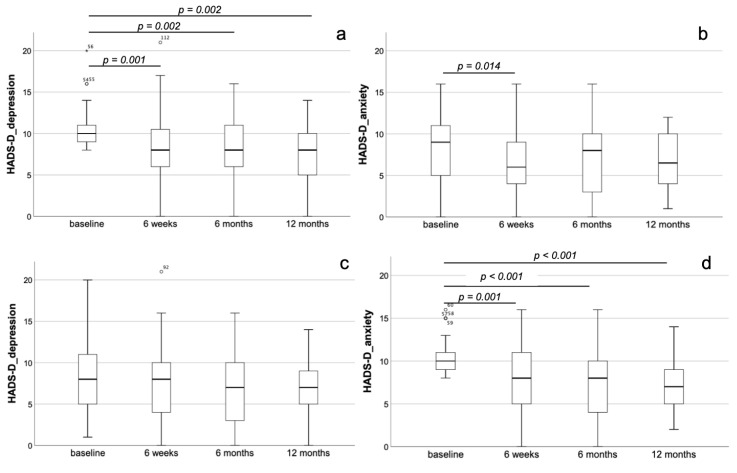
Dynamics of depression and anxiety after TAVI in the entire collective. Development of depression (**a**) and anxiety (**b**) in the depressive subgroup of the entire patients’ collective showing a significant improvement already 6 weeks after TAVI for both depression and anxiety. For depression but not for anxiety, this improvement remained stable even until the 6-month as well as the 12-month follow-up. Development of depression (**c**) and anxiety (**d**) in the anxious subgroup of the entire patients’ collective showing no significant changes with respect to depression but a significant reduction of anxiety 6 weeks after TAVI, remaining stable until the 6-month and the 12-month follow-up.

In the depressive subgroup, there was a significant improvement already 6 weeks after TAVI for both depression (8.3 ± 4.3 at the 6-week follow-up vs. 10.3 ± 2.5 at baseline, *p* = 0.001) and anxiety (6.3 ± 3.9 at the 6-week follow-up vs. 8.1 ± 3.9 at baseline, *p* = 0.014). For depression but not for anxiety, this improvement remained stable even until the 6-month (7.9 ± 3.8, *p* = 0.002 when compared with baseline) as well as the 12-month (7.7 ± 3.4, *p* = 0.002 when compared with baseline) follow-up (Figure 2a,b). Concomitantly, we observed significant improvements after 6 weeks for BNP (*p* < 0.001) and SMWT (*p* = 0.003). These changes were stable even until the 12-month follow-up for BNP (*p* < 0.001 compared to baseline) and SMWT (*p* = 0.023 compared to baseline). Additionally, there was an amelioration of EQ-5D-5L_anxiety/depression (*p* = 0.026) and EQ-VAS (*p* = 0.037) 6 months after TAVI, which was stable for EQ-5D-5L_anxiety/depression after 12 months (*p* = 0.024, each compared to baseline).

In the anxious subgroup, there were no significant changes with respect to depression (*p* = n.s. for all time-points), but a significant reduction of anxiety 6 weeks after TAVI (8.1 ± 3.8 at the 6-week follow-up vs. 10.4 ± 2.0 at baseline, *p* = 0.001), remaining stable until the 6-month (7.3 ± 3.8, *p* < 0.001 when compared with baseline) and the 12-month (7.2 ± 3.2, *p* < 0.001 when compared with baseline) follow-up (Figure 2c,d). As already shown for the depressive subgroup, we could observe a significant decrease of BNP after 6 weeks (*p* = 0.004), remaining stable also after 6 months (*p* = 0.002) and 12 months (*p* < 0.001, each compared to baseline). With respect to the remaining parameters assessed in the anxiety subgroup, there were significant improvements only in EQ-5D-5L_anxiety/depression (*p* = 0.036) and EQ-VAS (*p* = 0.039) after 6 weeks, which persist at the 6-month and 12-month follow-up (*p* < 0.05 for both compared to baseline). 

### 3.5. Predefined Subgroups According to Depression and Anxiety (HADS-D) Comparing Staging Group A and B

To investigate, whether the dynamics of depression and anxiety after TAVI are associated with the extent of extra-valvular cardiac damage according to the 2017 staging classification, the study collective was categorized as described above. Below, the portion of patients showing pathologic values for depression or anxiety both in staging group A (left heart damage) and B (additional pulmonary hypertension/right heart damage), have been analyzed to elucidate potential differences in treatment response. 

**Figure 3:**
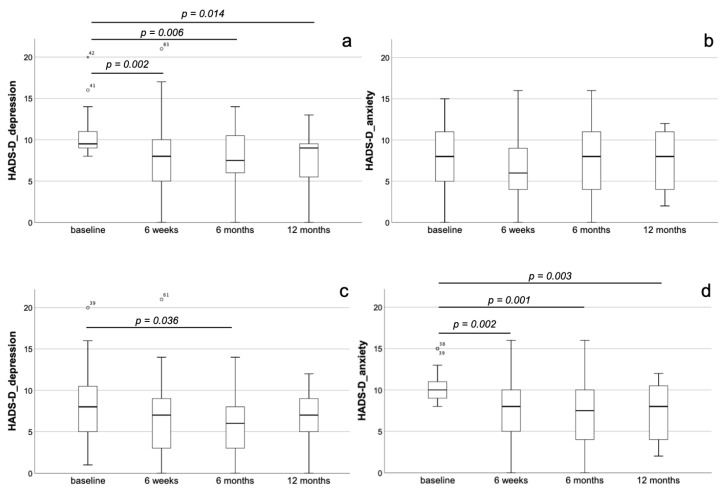
Dynamics of depression and anxiety after TAVI in staging group A. Development of depression (**a**,**c**) and anxiety (**b**,**d**) in the depressive (**a**,**b**) and the anxious (**c**,**d**) subgroup of staging group A. In the depressive subgroup, there was a significant and persisting decrease of depression at the 6-week, the 6-month as well as the 12-month follow-up, each compared to baseline (**a**). In contrast, in these patients, no dynamics with respect to anxiety could be observed (**b**). In the anxious subgroup, we could also detect a significant and stable decrease of anxiety at the 6-week, the 6-month and at the 12-month follow-up, each compared with baseline (**c**). When considering depression in these patients, we could not evidence any dynamics over time (**d**).

In staging group A, the totality of patients did not reveal relevant dynamics regarding the parameters of HADS-D at the different follow-up time-points (*p* = n.s.). In the depressive subgroup of staging group A, there was a significant and persisting decrease of depression at the 6-week (8.0 ± 4.5, *p* = 0.001), the 6-month (7.6 ± 3.5, *p* < 0.001) as well as the 12-month (7.7 ± 3.3, *p* < 0.001) follow-up, each compared to baseline (10.1 ± 2.4). In contrast, in these patients, no dynamics with respect to anxiety could be observed (*p* = n.s. for all follow-up time points) (Figure 3a,b). In the anxious subgroup of staging group A, we could also detect a significant and stable decrease of anxiety at the 6-week (7.9 ± 3.8, *p* = 0.002), the 6-month (7.4 ± 4.1, *p* = 0.001) and at the 12-month (7.2 ± 3.5, *p* = 0.003) follow-up, each compared with baseline (10.3 ± 1.9). Again, when considering depression in these patients, there was a significant decrease only when comparing the 6-month follow-up (5.9 ± 3.8) with baseline (8.1 ± 4.1, *p* = 0.036) (Figure 3c,d). Similar to the entire patients’ collective of the study (*n* = 224), also in staging group A (*n* = 158), a significant and stable improvement in EQ-VAS and SMWT as well as significantly lower BNP serum levels after TAVI could be proven (*p* < 0.05 for all follow-up time-points compared to baseline). 

**Figure 4:**
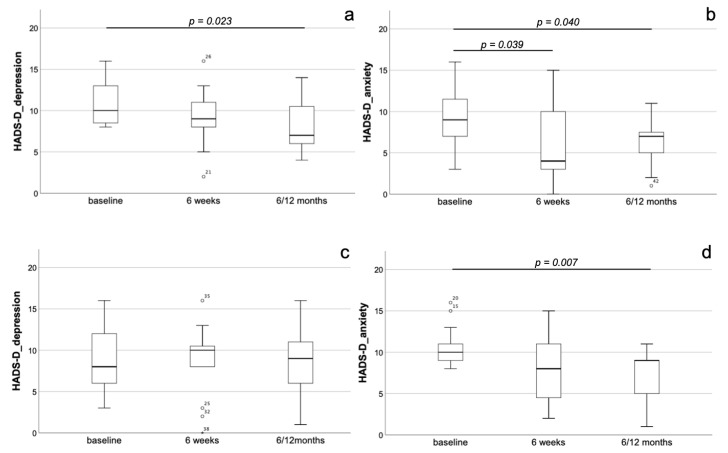
Dynamics of depression and anxiety after TAVI in staging group B. Development of depression (**a**,**c**) and anxiety (**b**,**d**) in the depressive (**a**,**b**) and the anxious (**c**,**d**) subgroups of staging group B. In the depressive subgroup, a relevant reduction in HADS-D depression values could not yet be detected at the 6-week follow-up but was present at the timepoint of long-term follow-up 6/12 months after TAVI compared to baseline (**a**). With respect to anxiety, there was an early and persisting decrease at the 6-week as well as the ‘long-term 6/12 months’ follow-up compared to baseline (**b**). In the anxious subgroup, there were no changes with respect to both depression and anxiety 6 weeks after TAVI. At the ‘long-term 6/12 months’ follow-up’, a significant reduction of anxiety but not depression could be observed compared to baseline (**c**,**d**).

As described for staging group A, also in the entire staging group B, we could not detect any changes in the parameters of HADS-D at the different follow-up time-points (*p* = n.s.). In the depressive subgroup of staging group B, a relevant reduction in HADS-D depression values could not yet be detected at the 6-week follow-up (9.0 ± 3.6, *p* = n.s. compared to baseline) but was present at the time-point of long-term follow-up 6/12 months after TAVI (8.1 ± 3.2, *p* = 0.023) compared to baseline (10.9 ± 2.7). In contrast to those of staging group A, the depressive patients of group B also showed an early and persisting decrease of anxiety measured by HADS-D at the 6-week (5.8 ± 4.5, *p* = 0.039) as well as the ‘long-term 6/12 months’ (6.2 ± 2.9, *p* = 0.040) follow-up compared to baseline (9.3 ± 3.9) (Figure 4a,b). In the anxious subgroup of staging group B, there were no changes in HADS-D parameters, both anxiety and depression, 6 weeks after TAVI (8.3 ± 3.9, *p* = n.s. compared to baseline). At the ‘long-term 6/12 months’ follow-up’, a significant reduction of anxiety (7.4 ± 3.0, *p* = 0.007) could be observed compared to baseline (10.4 ± 2.3). In contrast, there were no dynamics with respect to depression in these patients (*p* = n.s.) (Figure 4c,d). With respect to the remaining parameters assessed in the study, in staging group B (*n* = 66), a significant and stable decrease of BNP serum levels and an improvement of the SMWT could be proven (*p* < 0.05 for all follow-up time-points compared to baseline).

## 4. Discussion

The impact of mental disorders, in particular depression and anxiety, as co-morbidities in elderly patients suffering from cardiovascular diseases, e.g., aortic stenosis, is not sufficiently investigated until now, but might be of great importance due to high estimated rates of unreported cases [23]. The majority of available studies in the field have focused on heart failure patients and could detect high prevalence rates for both depression and anxiety. Inclusion criteria and description of heart failure presentation with respect to morphology, function and underlying etiology are very heterogeneous in these studies. Thus, it is challenging to draw robust conclusions, whether there is a link between the objective extent of cardiac damage and dysfunction, as assessable by established imaging modalities, e.g., echocardiography, and severity of mental disorder symptoms [14]. Our elderly AS patients’ collective represented a typical TAVI collective exhibiting moderate surgical risk with an STS score that was mildly below the score of the large PARTNER 2 trials. The 224 patients included by us were classified according to the 2017 staging classification and the distribution was quite similar to that in the PARTNER 2 trials collective, in which it could be shown to be of prognostic relevance [24]. We detected a significant and stable decrease in BNP serum levels and an improvement in the SMWT during the 1-year follow-up period after TAVI. This observation reflects treatment success and goes in line with the findings of nearly all large TAVI trials irrespective of surgical risk [6,7,8,11,12]. 

Using the HADS-D questionnaire, we could detect a prevalence rate of 25.4% for depression and 26.8% for anxiety, which is rather high and corresponds to data reported in recent studies. In the entire collective, there were no changes with respect to the mean HADS-D depression or anxiety score values up to 1 year after TAVI. In contrast, when analyzing the subgroups of patients classified as depressive or anxious prior to TAVI, there were significant and stable improvements for the respective item. These findings go in line with a recent study of our group [23]. In particular, with respect to depressive symptoms, also in heart failure patients, a relevant treatment-associated improvement has been reported recently [30,31]. Since there is a significant and stable decrease in BNP serum levels in the entire collective as well as in the depressive and anxious subgroups, the mental improvements might not simply reflect the improvement of heart failure after TAVI. This corresponds to a recent study in heart failure patients, in which there was no association between severity of depressive symptoms and BNP serum levels [32]. As in the entire collective, also in patients of the depressive subgroup, there was a significant and stable improvement in SMWT in our study not detectable in the anxious subgroup. Similar observations have been reported from the SADHART-CHF trial, in which there was an improvement in SMWT in heart failure patients showing treatment-associated depression remission compared to the non-remission group [33]. With respect to anxiety, studies in the field are rare. Nevertheless, in contrast to depression, which was independently associated to the SMWT in heart failure patients in a recent trial, such a correlation has not been observed for anxiety [34]. In our current study, we could not show, for the first time, significant differences between the two staging groups of extra-valvular cardiac damage extent with respect to the distribution of patients showing pathologic values for neither depression nor anxiety as assessed by HADS-D prior to TAVI.

In that context, missing correlations between the severity of mental disorder-associated symptoms, in particular depression, and the extent of structural and functional cardiac impairment have been reported in a variety of studies [35]. Thus, when comparing severely depressive with non-depressive heart failure patients, Aguiar and co-workers could not observe clinically relevant differences regarding left ventricular ejection fraction (LVEF) [36]. Also, after acute myocardial infarction, the presence of depression was not significantly associated with LVEF in a very recent trial [37]. With regard to potential differences between heart failure with reduced ejection fraction (HFrEF) and preserved ejection fraction (HFpEF), Kato and colleagues described similar rates of depression, which was 24% in HFrEF and 25% in HFpEF, respectively. As already shown in many heart failure studies, also here, the presence of depression could be shown to be a robust predictor of worse outcome in HFrEF and HFpEF, independent of BNP serum levels [38]. Interestingly, depression also predicts death and rehospitalization irrespective of NYHA stage or heart failure severity measured by BNP [39,40]. While, in contrast to depression, for anxiety, the majority of studies did not report any effect on outcome and prognosis, Lin and colleagues observed the notable fact that anxiety was independently associated with mortality and rehospitalization in HFpEF but not HFrEF patients [41].

When comparing the two staging groups defined in this study, the only difference with respect to the dynamics of depression and anxiety in the subgroups showing pathologic values prior to TAVI was the phenomenon that in staging group B compared to A, the improvement of depression and anxiety symptoms was timely delayed. 

Since the idea of the staging classification of AS-associated extra-valvular cardiac damage is rather new, there is a very limited number of available studies for discussing this finding. In a recent large trial, including 9566 patients that survived myocardial infarction in England, a clear association between reduced QoL using the EQ-5D questionnaire, which includes an anxiety/depression item, and the presence of further chronic disease, e.g., chronic obstructive pulmonary disease, could be shown [42]. Against that background, our finding of a delayed recovery of depressive or anxious AS patients in staging group B appears to be comprehensible. Another interesting study partially supporting our findings investigated QoL and depression in a 2-year follow-up among patients referred for heart transplant. When comparing the 3 groups of transplant recipients, transplant candidates and medically stable heart failure patients, there were higher scores of mental health and less depressive symptoms in the medically stable compared to the other groups, speaking well for the fact that mental recovery is delayed in patients showing either persisting somatic illness, like those of the transplant candidates group, or additional complex co-factors affecting mental health, which are easily conceivable in the transplant recipient group [43]. Another aspect likely explaining our findings is the fact that pulmonary hypertension and/or right heart failure are determinants of staging group B in our AS patients. Not only for group 2 pulmonary hypertension (due to left heart disease), which is the most common variant in our patients, but also for other forms, a significantly reduced QoL has been proven [44], in particular when being accompanied by right heart failure [45].

A potential bias when investigating dynamics of depression and anxiety in follow-up after interventional procedures, e.g., TAVI, is the occurrence of peri-procedural complications [46,47]. Thus, severe complications, which are very rare in our local registry, were excluded from our analysis. 

Taken together, although higher stages of extra-valvular cardiac damage in AS patients are of proven prognostic relevance, we did not observe an association with higher rates of pre-existing depression or anxiety. The TAVI procedure resulted in a persisting reduction of depression and anxiety in patients showing pathologic values at baseline. Notably, these improvements are timely delayed in higher stages.

## Figures and Tables

**Table 1:** Baseline characteristics of the patients included in the study: entire collective and predefined staging groups A and B.

Parameter	All Patients (*n* = 224)	Staging Group A (*n* = 158)	Staging Group B (*n* = 66)	*p*-Value (Staging Group A vs. Group B)
**Age (years)**	77.9 ± 7.5	77.5 ± 7.5	78.7 ± 7.5	n.s.
**Male (%)**	45.1	44.3	47	n.s.
**STS score**	4.4 ± 3.1	3.9 ± 2.5	5.5 ± 3.8	0.005
**NYHA stage > II (%)**	72.4 (*n* = 221)	70.7 (*n* = 157)	76.6 (*n* = 64)	n.s.
**Angina pectoris (%)**	30 (*n* = 223)	31	27.7 (*n* = 65)	n.s.
**CAD (%)**	61.6	56.3	74.2	0.012
**PAD (%)**	12.5	12	13.6	n.s.
**Diabetes (%)**	41.7	44.9	34.8	n.s.
**COPD (%)**	18.3	20.9	12.1	n.s.
**Atrial fibrillation (%)**	46.6 (*n* = 223)	35 (*n* = 157)	74.2	<0.001
**Pacemaker pre TAVI (%)**	13.4	12.7	15.2	n.s.
**GFR ≤ 30 mL/min. (%)**	16.1	13.9	21.2	n.s.
**BNP ≥ 100 (%)**	90.2 (*n* = 205)	87.4 (*n* = 143)	90.9 (*n* = 62)	0.038
**LVEF (%)**	58.6 ± 14.3 (*n* = 221)	60.7 ± 13	53.6 ± 16	0.001
**Mitral regurgitation ≥ II° (%)**	36.5 (*n* = 222)	26.9 (156)	56.1	<0.001
**Tricuspid regurgitation ≥ II° (%)**	24.9 (*n* = 221)	0	78.8	<0.001
**Stage 0–4 (%)**				
**0**	2.7	3.8	-	
**1**	12.5	17.7	-	
**2**	55.4	78.5	-	
**3**	18.8	-	63.6	
**4**	10.7	-	36.4	
**Edwards Sapien 3 (%)**	62.9	63.3	62.1	n.s.
**CoreValve Evolut (%)**	36.2	36.1	36.4	n.s.
**Acurate neo (%)**	0.9	0.6	1.5	n.s.
**HADS-D_depression ≥ 8 (%)**	25.4	26.6	22.7	n.s.
**HADS-D_anxiety ≥ 8 (%)**	26.8	24.7	31.8	n.s.

Abbreviations: STS—Society of Thoracic Surgeons; NYHA—New York Heart Association; CAD—coronary artery disease; PAD—peripheral artery disease; COPD—chronic obstructive pulmonary disease; GFR—glomerular filtration rate; BNP—brain natriuretic peptide; LVEF—left ventricular ejection fraction; TAVI—transcatheter aortic valve implantation; n.s.—non-significant.

## Data Availability

The data presented in this study are available in this manuscript.
